# Bringing Transdisciplinary Aging Research From Theory to Practice

**DOI:** 10.1093/geroni/igab046.2013

**Published:** 2021-12-17

**Authors:** Lana Sargent, Tracey Gendron, Marissa Mackiewicz, Ana Diallo, Faika Zanjani, Elvin Price, Pamela Parsons, Gregory Ford

**Affiliations:** 1 VCU School of Nursing, Ashland, Virginia, United States; 2 Virginia Commonwealth University, Richmond, Virginia, United States; 3 VCU Institute for Inclusion, Inquiry, and Innovation (iCubed): Health and Wellness in Aging Populations, Richmond, Virginia, United States; 4 Virginia Commonwealth University School of Nursing, Richmond, Virginia, United States; 5 Virginia Commonwealth University/ School of Pharmacy, Richmond, Virginia, United States; 6 Beacon Housing Communities, Richmond, Virginia, United States

## Abstract

There is a growing emphasis to use a transdisciplinary team approach to accelerate innovations in science to solve complex conditions associated with aging. However, the optimal organizational structure and process for how to accomplish transdisciplinary team science are unclear. In this study, we illustrate our team’s experience using transdisciplinary approaches to solve challenging and persistent problems for older adults living in urban communities. We describe our challenges and successes using the National Institutes of Health four-phase model of transdisciplinary team-based research. Using a de-identified survey, the team conducted an internal evaluation to identify features that created challenges including structural incongruities, interprofessional blind spots, group function, and group dynamics. The team then identified responses to address the features that created challenges and determined indicators for success. Indicators for success were identified by the team as a place for continued evaluation of the teams’ collaborative effectiveness, transdisciplinary integration, and impact on the university and aging community. This work resulted in the creation of the team’s Transdisciplinary Conceptual Model. This model became essential to understanding the complex interplay between societal factors, community partners, and academic partners. Conducting internal evaluations of transdisciplinary team processes is integral for teams to move beyond the multi- and interdisciplinary niche and to reach true transdisciplinary success. More research is needed to develop measures that assess team transdisciplinary integration. Once the process of transdisciplinary integration can be reliably assessed, the next step would be to determine the impact of transdisciplinary team science initiatives on aging communities.

